# Opportunistic Pathogens of Recreational Waters with Emphasis on Antimicrobial Resistance—A Possible Subject of Human Health Concern

**DOI:** 10.3390/ijerph19127308

**Published:** 2022-06-14

**Authors:** Joanna Stec, Urszula Kosikowska, Mariola Mendrycka, Dagmara Stępień-Pyśniak, Paulina Niedźwiedzka-Rystwej, Dominika Bębnowska, Rafał Hrynkiewicz, Joanna Ziętara-Wysocka, Ewelina Grywalska

**Affiliations:** 1 Department of Pharmaceutical Microbiology, Medical University of Lublin, 20-093 Lublin, Poland; madurajoanna@gmail.com (J.S.); urszula.kosikowska@umlub.pl (U.K.); 2 Department of Nursing, Kazimierz Pulaski University of Technology and Humanities in Radom, 26-600 Radom, Poland; m.mendrycka@uthrad.pl; 3 Department of Veterinary Prevention and Avian Diseases, Institute of Biological Bases of Animal Diseases, Faculty of Veterinary Medicine, University of Life Sciences in Lublin, 20-950 Lublin, Poland; dagmara.stepien@up.lublin.pl; 4 Institute of Biology, University of Szczecin, 71-412 Szczecin, Poland; paulina.niedzwiedzka-rystwej@usz.edu.pl (P.N.-R.); rafal.hrynkiewicz@usz.edu.pl (R.H.); 5 Provincial Sanitary and Epidemiological Station in Szczecin, 71-899 Szczecin, Poland; joannaz80@wp.pl; 6 Department of Experimental Immunology, Medical University of Lublin, 20-093 Lublin, Poland; ewelina.grywalska@gmail.com

**Keywords:** water-derived opportunistic pathogens, water contamination, risk factor, physical activity, water users health

## Abstract

Infections caused by exposure to opportunistic pathogens can cause serious health problems during recreational water use. The problem of diseases caused by microbes transmitted by water is a major public health challenge, especially in developing countries with economic problems and poor hygiene conditions. Moreover, the quality of water in natural reservoirs is often at a very low level in terms of microbiological water purity, which means that their use for recreational purposes, but also as a source of drinking water, may have serious health consequences. Recreational waters pose a threat to human health. Therefore, the quality of recreational waters is closely monitored in many jurisdictions. In this review, we summarize key information on the most common pathogens that can be water-based or waterborne. The issue of antimicrobial resistance among opportunistic pathogens remains equally important. It is important not only to fight pathogens, but also to take action to reduce chemical stressors (especially antibiotics) in the aquatic environment, and to understand the various mechanisms of the spread of antibiotic-resistant genes.

## 1. Introduction

The safe and accessible supply of drinking as well as recreational water should be available to all because both can result in significant benefits to health. A public health problem in developing countries, mainly among people with the lowest economic conditions and the poorest hygienic facilities, are etiological factors of waterborne microbial diseases. For many researchers and practical users, the crucial thing is to consistently ensure the safety of drinking water at all stages of the water supply, from the catchment area to the consumer [1]. Opportunistic bacteria and other microorganisms isolated from man-made and natural recreational water reservoirs create possibilities of associated infections or other diseases. The human health risk associated with non-potable water, especially from natural sources, is still uncertain. Surface water quality is subject to fast, dramatic, and dangerous changes in microbial quality as a result of a variety of activities of humans and animals. These changes are caused by the release of municipal wastewater and runoff of rainwater, along with pollution from farmlands and animal husbandry to water bodies such as rivers and lakes [1,2]. Meanwhile, in many countries natural water reservoirs or wild waters are often used for a public utility, particularly for sources of potable or cleaning water (e.g., African countries) and for recreational use (e.g., European countries) [2]. Recreational use of water means, e.g., immersion of a body or a head, splashing the water, swimming, wading, and finally, swallowing the water accidentally [3]. Water reservoirs can be divided into natural (e.g., fresh and coastal waters) and artificial (e.g., pools and spas). They can be a source of opportunistic microorganisms including waterborne pathogens.

The term “waterborne” pathogens is used to denote pathogens of fecal origin. There are also pathogens which, due to their non-canal origin, are referred to as “water-based”, and these pathogens are also referred to as opportunistic. It is also important that water-based pathogens grow and develop in natural and artificial water reservoirs [4]. Additionally, environmental water is not often a natural habitat for many opportunistic microorganisms (e.g., enterococci) and their presence in this milieu may be considered as a consequence of, e.g., fecal pollution [5]. Water-transmitted microorganisms and water-related diseases are the most important cause of morbidity and mortality worldwide [6]. These microorganisms, e.g., bacteria, fungi, and parasites can create opportunistic infections, which are defined as infections that occur more often or are more severe in people with weakened immune systems than in people with healthy immune systems [7]. On the basis of some investigations the health effects of recreational exposure to various sources of water are associated with opportunistic pathogens [8]. There is little information on the statistics of diseases caused by poor microbiological conditions of bathing areas, especially those used only occasionally, e.g., during the summer period. According to the WHO, more than 50% of water-related-disease mortality is caused by bacterial intestinal infections [9]. This may be due to the long exposure to a microbial risk before it is detected or low pathogenicity known for opportunistic pathogens. That is why it is so important to constantly monitor the quality of recreational waters so as not to pose a health risk to people [10]. However, it should be remembered that in the context of recreational bathing, water is not the only risk, as there are also other vectors that carry pathogenic microorganisms. Admission to the resorts and swimming pools is often paid. Recent studies indicate that contaminated banknotes can bridge the transmission of pathogens between people, as the microorganisms contaminating paper money are highly persistent and bacteria can be easily removed and transferred to the skin or other objects [11].

The microbiota residing in the various sources of water as well as water distribution systems can have an effect on their quality, especially in drinking water but also in recreational use of water. Besides, bacterial community composition could have a link with the occurrences of opportunistic pathogens important for human health. The most important fecal-indicator bacteria present in drinking water are discussed, focusing on the limitations of their use as markers in other types of water important for human life. *Escherichia coli* and *enterococci* are two fecal indicator organisms (FIO) widely used to monitor the quality of recreational waters [12,13]. As they are both present in human and animal feces, they are called bacterial fecal-contamination indicators. The presence of *Enterococcus* spp. can indicate possible long-standing fecal contamination of water.

The purpose of this review is to present the health risks associated with microorganisms that cause water-related diseases for humans. A search of studies published was conducted based on literature and website data of harmful human health effects after contact with microorganisms and microbiologically contaminated water. The characteristics most important for human health, bacteria occurring in water and transmitted by water, and the infectious diseases caused by them, are presented. The importance of antimicrobial resistance by opportunistic pathogens and their participation in diseases occurring after recreational contact with water in places intended for bathing were also discussed. Finally, we want to briefly discuss the law regulations regarding the monitoring of the quality of recreational waters.

## 2. Opportunistic Pathogens in Recreational Water

### 2.1. Sources of Water Contamination

Although many microorganisms are naturally present in the water, there are different sources of this habitat pollution. According to varied epidemiological studies, water pollution is divided into known point (e.g., of human fecal pollution) and non-point sources [3]. The non-point sources of pollution include sand re-suspension, animal feces, storm water runoff, and human skin microorganisms. These sources may contain human fecal input. However, it is difficult to distinguish whether pollution is caused by animals or humans in cases of non-point source inputs [3]. Remarkably, many opportunistic pathogens possess the capacity to survive and proliferate in water-based environments, such as fish and other water bodies, natural water sources, lakes, rivers, and liquids containing small amounts of nutrients [14]. These conditions can create microbial exposure and contamination episodes associated with the recreational and bathing activity and ability of microorganisms to live in environmental water.

Animal input may be caused mainly by dogs, birds, pigs, and cows while they are near or in the water [15]. Epidemiological studies reviewed by Dufour et al. stated that there was no association between exposure to recreational water polluted by non-human feces and swimming-associated gastrointestinal illness [16]. Animal input can increase the fecal indicator load [3]. Gulls are important contributors of fecal contamination to surface waters and recreational beaches [17]. What is more, human pathogens, such as *Salmonella* spp., *Campylobacter* spp., or *E. coli* have been detected in gull fecal samples. Converse et al. demonstrated that gull removal (using specially trained dogs) contributed significantly to improvement of water quality [17]. On the other hand, Wang et al. describe that gulls contribute to the fecal load relatively little. They indicate that dogs are responsible for the larger source of enterococci relative to human and birds [18].

One of the most important sources of bacteria, especially multidrug-resistant bacteria, that contaminate the environment is animal manure and slurry from large-scale farming of consumer animals (poultry, swine, cattle) that are used to naturally fertilize fields. Pathogens, including opportunistic ones, present in manure and slurry can spread in soil and aquatic environments and remain infectious for long periods of time.

In addition, both manure and slurry often contain drug residues or their metabolites, further enhancing the selection of bacterial resistance in the environment. Excessive use of crop protection products also plays a major role in the development of bacterial resistance. Drug-resistant bacteria, antibiotic metabolites, and plant protection products contaminate surface and groundwater.

The list of bacteria whose appearance in manure and slurry can pose a serious threat to humans and animals, under European conditions, includes, e.g., *Brucella* spp., *Chlamydia* spp., *E. coli* (enteropathogenic antibiotic-resistant strains), *Leptospira* spp., *Rickettsia* spp., *Salmonella* spp., *Treponema hyodysenteriae*, *Bacillus anthracis*, *Erysipelothrix rhusiopathiae*, *Mycobacterium* spp. Their presence and number depends on environmental factors, the animal species from which the manure and slurry originates, and its physicochemical properties and composition [19]. The circulation of antibiotic-resistant bacteria in the environment poses a risk of infecting humans with them. From the data of the European Food Safety Authority (European Food Safety Authority-EFSA) shows [19] that the most common cause of decreasing effectiveness of antibiotic therapy is infections caused by antibiotic-resistant bacteria of the genera: *Salmonella*, *Campylobacter*, *Enterococcus,* and some serotypes of *Escherichia coli* species [14].

Microorganisms inhabiting surface of a body, stirred polluted sand or sediment may constitute source of water pollution. It is assumed that gastrointestinal disorders and respiratory pathogens are transmitted by bather to bather via the water and air [3,20].

People’s awareness of the type and source of pollution is very important, especially the fact that the main cause of its creation is destructive human activity. Apart from humans and wild animals, excrements of domestic animals such as dogs or cats are also a threat to the environment, posing a significant source of threat to the quality and microbiological purity of naturally occurring waters.

### 2.2. Waterborne and Water-Based Opportunistic Pathogens in Recreational Water

There are some pathogens of non-fecal origin that can be transmitted via water, such as *Legionella pneumophila*, *Mycobacterium avium complex,* and *Pseudomonas aeruginosa* [3,4]. According to Pruden et al., the pathogens mentioned above should be called ‘water-based’ instead of ‘waterborne’, because pathogens of fecal origin are called ‘waterborne’. Water-based microorganisms are also called ‘opportunistic pathogens’. Water-based pathogens generally grow and thrive both in natural and engineered water systems [4].

Ingestion of certain pathogens does not always have the most detrimental effect on human health. In some cases, infections after inhalations of aerosols containing *Legionella* spp. or skin colonization by *P. aeruginosa* appear to be more dangerous [4]. Currently, the provision of clean drinking water through well-carried-out water treatment processes reduces the transmission of pathogens to humans.

Pruden’s classification of pathogenic microorganisms according to their mode of transmission to humans is important in identifying the cause of ailments. Only some people associate their symptoms with contaminated water if they have not come into direct contact with it at all. Knowing that harmful microorganisms can enter the body not only through direct immersion in water, but also through inhaled aerosols (e.g., *Legionella pneumophila*), can positively influence the conversation with the doctor and result in a quicker diagnosis. This is of great importance in developing a treatment regimen and quickly stopping the adverse growth of microorganisms in the body.

Recreational use of water means, e.g., immersion of a body or a head, splashing the water, swimming, wading, and finally, swallowing the water accidentally [3]. Water reservoirs can be divided into natural (e.g., fresh and coastal waters) and artificial (e.g., pools and spas). They can be a source of waterborne pathogens. Additionally, environmental water is not often a natural habitat for many opportunistic microorganisms (e.g., enterococci) and their presence in this milieu may be considered as a consequence of, e.g., fecal pollution [5]. The transport and contamination of opportunistic pathogens from natural reservoirs of surface water to groundwater increases a serious risk in resources of water [21]. As has been demonstrated in vitro with Gram-negative bacteria *Burkholderia cepacia* complex the exchange of genetic material between different strains/species living in water might occur, creating the possibility for apparently innocuous strains to acquire pathogenic features [22]. Environmental microorganisms often have the ability to rapidly adapt through various ways including, e.g., mutation, which translates into a remarkable genotypic and phenotypic plasticity and their metabolic diversity and capacity to endure environmental stresses [23,24]. Consequently, water-transmitted microorganisms and water-related diseases are a very important cause of morbidity and mortality worldwide [6,25]. According to WHO [26,27], each year, 3.4 million people, mostly children (about 1.4 million ones), die from low water quality and water-related diseases.

A range of disease-causing microorganisms can be present in water, including examples listed in Table 1. Bacterial inhabitants of waterbodies can colonize both people and animals, as well as various water systems. Longer duration of activity means a greater degree of exposure to a higher risk for water-related diseases [3].

### 2.3. Water as a Source of Disease-Causing Microorganisms

Many forms of water activities can contribute to wide range of diseases, including skin and soft tissue infections, gastrointestinal illnesses, respiratory tract infections, acute febrile illnesses, and even central nervous system infections [28]. Recreational activities can take many forms, such as swimming, diving, rafting, kayaking, fishing, waterskiing, and other water sports. Such activities can lead to exposure to various microorganisms in the water. Considering the fact that nowadays people tend to travel and use natural reservoirs for bathing, this involves more activity in freshwater, which in turn leads to an increased risk of infection. Sometimes those infections are not considered to be acquired through travel or recreational activity. Perhaps a medical interview after travel should be collected from returning travelers who exhibit specific symptoms [28].

Water may act as a reservoir of specific pathogens and opportunistic microorganisms, which are a cause of infections (Table 2). Water that includes *E. coli* and *enterococci* can pose a threat of gastrointestinal illness to water recreators [5]. The concentration of fecal indicator (e.g., *E. coli*) in surface water may be associated with a continuum of symptom severity among water recreators [5].

Gastrointestinal illness is defined as diarrhea, vomiting, or stomach pain that interferes with daily activities, or nausea that accompanies stomach pain or disturbs daily activities [5]. Currently, there is no appropriate definition for gastrointestinal infection symptoms resulting from waterborne infections. Characterization of symptom severity and attempts to evaluate measures of them can contribute to a better understanding of the risks, predictors, and consequences of recreational waterborne illness.

People can acquire infections transmitted via water through a variety of ways. The most common way is ingesting fecally contaminated food or water [28]. According to recreational activities, swallowing water while swimming and freshwater recreation are important risk factors for human health. Besides leptospirosis infection by ingestion, it is also possible to infect through contact with contaminated water with non-intact skin [28]. The risk of skin and soft-tissue infections can increase when the injury occurs. As an example, *M. marinum* infection after trauma can be a result of previous body contact with fish or shellfish [28]. *Cryptosporidium* is a protozoan parasite which can survive in the environment even after several months and is very easily transmissible to the next host. In the case of cryptosporidiosis, a very small infectious dose is required to establish infection [29]. In addition to this, free-living amebas infections, such as *Naegleria fowleri*, start with the inhalation of contaminated water with the nasal cavity [28].

The incidence of health consequences after water recreation activities such as gastrointestinal illness, upper respiratory tract infections, rash, earache, or eye irritations were lowest among people aged 55 and older [35,36]. The detrimental effect on human health was noticed more often among children. The reason may be that adults swallow less water while swimming than children [37,38]. Older people are less likely to experience symptoms because they tend to be less exposed to water during recreational activity [39]. Thus, it is obvious that the detrimental effects on human health depend on the amount of water swallowed, not the age of the person exposed to the microbes.

*P. aeruginosa* can be an etiological factor of folliculitis in the elderly. ‘Hot tub’ or ‘spa pool’ folliculitis occurs when many people use the tub or pool and the water is poorly chlorinated. There are several sources of such infection—swimming pools, poorly cleaned or contaminated bathtubs, or sponges [40,41].

The infectious dose of pathogens means the number of organisms needed to infect sensitive individuals (Table 2).

The risk of occurrence and spreading infections in humans depends on exposure to different sources of aquatic pathogenic microorganisms, including opportunistic pathogens. Among aquatic pathogens there are some bacterial species of the genera *Aeromonas*, *Shigella*, *Salmonella*, *Campylobacter*, *Legionella,* and other Gram-negative bacilli [3,18]. The known water-connected diseases with casual bacterial agents are, e.g., cholera (*Vibrio cholerae*), bacillary dysentery or shigellosis (*Shigella dysenteriae*, *S. flexneri*, *S. boydii*, *S. sonnei*), typhoid fever and other serious salmonellosis (*Salmonella enterica* subsp. *enterica* serovars Paratyphi, Typhi or Typhimurium), acute diarrheas, and gastroenteritis (*E. coli*, particularly serotypes such as O148, O157 and O124) [39]. Besides, Gram-positive bacteria such as *Enterococcus* spp. or *Staphylococcus* spp. were selected as common pathogens of water-connected opportunistic infections. All these infectious bacteria contain species that may cause serious infectious diseases (Table 2). Moreover, the consequences for human health are strongly related to the individual’s health condition. For example, compared with healthy people, the infectious dose for opportunistic pathogens is lower for immunocompromised people or those treated with antimicrobials, e.g., antibiotics [3]. However, the risk of infection from swimming in natural recreational water sources is still uncertain. The important problem is that the principal habitat of microorganisms such as, e.g., *Shigella* spp. or *Salmonella* spp. is the intestinal tract of humans and animals [40]. Many pathogenic bacteria are excreted by humans, pets, and farm animals with feces, and are constantly found in environmental samples, including water (Table 3). The main sources of these pathogens in natural environments, including recreational waters, may be, e.g., municipal sewage, agriculture pollution, and storm water runoff [42,43]. These bacteria can survive for a long time in water and in soil if conditions of humidity, temperature, and pH are appropriate [40]. For this reason, it is so important to use natural water reservoirs rationally and carefully for recreational purposes.

## 3. Antimicrobial Resistance of Microorganisms Isolated from Water

Early and appropriate antibiotic treatment is important and can shorten the course of infections. To ensure the correct treatment of waterborne/water-based infections, the etiological agent should be identified. Patients with bacterial infections can be treated with proper antibiotics directed to the identified cause. Moreover, more extensive infections following recreational water exposure may require systemic antibiotic therapy.

Various sources of bacterial water pollution (e.g., fecal or from agricultural sources) and many factors have an influence on the phenomenon of increasing bacterial resistance [4]. This is why a wide range of antibiotic classes may be useless in treating life-threatening bacterial infections in the face of the increasing resistance phenomenon. Although, clinicians know both the resistance and pathogenic mechanisms of famous pathogens such as *E. coli* or *Salmonella* spp, they still have problems with curing opportunistic infections. It is important that opportunistic pathogens should be taken into consideration while creating new treatment guidelines. They represent a poorly understood source of infections, as well as a source of resistance genes because of their remarkable ability to donate genes. Moreover, the problem of antibiotic resistance among microorganisms may become the leading cause of death by 2050 [51]. Among microorganisms isolated from water reservoirs, antimicrobial resistance, including multiple antibiotic resistant (MAR) human pathogens (e.g., *L. pneumophila*, *P. aeruginosa* and *Aeromonas* species) was observed [4].

Antibiotic resistance is a serious challenge as physicians usually treat bacterial infections using empirical therapy without further verification by microbiological tests. They make decisions based on epidemiological data and their own observations in their patient population. Unfortunately, their decisions are not always appropriate. What is more, tightening of the rigor associated with prescribing antibiotics and improvement of the sanitation status were intended to help with limiting the spreading of resistance [52]. The WHO Global Action Plan for Combatting Antibiotic Resistance specified that a key linkage and crucial point in reducing antimicrobial resistance is water, hygiene rules, effective sanitation systems, and infection prevention [52]. Unsanitary living conditions contribute to rapid person-to-person infection spreading. Investment in water and sanitation infrastructure can help with slowing the drug resistance [51].

There are three principal ways that antibiotics reach the environment: animal waste, human waste, and manufacturing waste [51]. It is hard to predict their degradation rate in the natural environment. When it comes to solution of the human and animal waste problem, it is crucial to reduce the inappropriate use of antibiotics. Due to raised public awareness, limiting the use and sale of products containing antimicrobials can be achieved. Environmental reservoirs of antibiotic-resistant bacteria can be created by releasing active pharmaceutical ingredients, by inappropriate treatment of waste products. This situation is especially harmful to people who are exposed to polluted water by living near manufacturing sites. The introduction of specific regulatory standards all over the world may minimize the amount of active pharmaceutical ingredients releasing into the environment. It is important to enforce manufacturer compliance with this law, especially in the locations such as China or India because these are places where companies can bear lower costs of production [51].

The Centers of Disease Control and Prevention (CDC) classify microorganisms into specific groups depending on their public health threats potential [53]. Among urgent threats that require aggressive action, CDC counts carbapenem-resistant *Acinetobacter* spp., *Clostridioides* (formerly *Clostridium*) *difficile*, and carbapenem-resistant *Enterobacteriaceae*. Mobile genetic elements such as insertion sequences, transposons, and integrons can be carried and easily shared by those bacteria. This makes some antibiotics ineffective and contributes to the spread of resistance to these important drugs. Such dangerous pathogens present in the recreational water may contribute to the development of pneumonia, wound, bloodstream, and urinary tract infections, or life-threatening diarrhea [53].

### Health Problems after Contact with Microbial-Contaminated Water including Drug-Resistant Bacteria

Cases of health problems caused after contact with contaminated water during recreational activities are not frequently reported. Reports or scientific works mainly concern the occurrence of *E. coli* and *Enterococcus* spp. and what is in accordance with legal requirements [54]. There are few reports relating to a problem with microbiological contamination spreading by water. According to the research conducted by Wolny-Koładka and Lenart-Boroń [55], 10% of *E. coli* strains isolated from the water reservoir were resistant to five antibiotics, 12% presented resistance to two or three antibiotics at the same time [55]. What is more, 4% of isolates were resistant to twelve antibiotics and 7% to nine antibiotics. Even though it does not seem to be a high percentage, it still can pose a serious threat, causing infection of recreational water users. Furthermore, reported cases usually present drug resistance to different antimicrobials, including the most popular antibiotics such as β-lactams [55]. This phenomenon is worrisome because there are many ways of spreading antimicrobial resistance (Table 4).

One of the most important mechanisms of drug resistance is the production of extended spectrum β-lactamases (ESBL). This mechanism is a common cause of *E. coli* resistance to penicillins, cephalosporins, and monobactams [60,61]. Infections of ESBL-positive strains mainly include urinary tract infections and nosocomial infections. Additionally, humans or animals can carry ESBL-positive microorganisms [55,60].

According to Wolny-Koładka and Lenart-Boroń (2016), although they did not observe extended spectrum β-lactamases-type resistance mechanisms phenotypically, the genes responsible for its emergence were detected in isolates derived from a reservoir [53]. This suggests that antimicrobial resistance can be encoded by multiple genes, or some genes were not expressed. What is more, in this publication, 74% of isolated strains were resistant to one or more antibiotics. The level of resistance to the tested drugs was much higher among strains isolated in summer in comparison to strains from winter. Nevertheless, the prohibition of bathing and swimming for people as well as animals in this reservoir in the summer increases the risk of serious infections.

Giebułtowicz et al. describe that among analyzed *Enterobacteriaceae* strains, β-lactams resistance, especially in subgroups of these antibiotics, was most frequently observed [59]. The high frequency of resistant microorganisms was caused by the discharge of sewage into the river, which may indicate only partial purification during the wastewater treatment process. The occurrence of high concentrations of azithromycin in the river was worrisome. This substance and other macrolide antibiotics are present on the watch list of substances that may pose an environmental risk and require monitoring (Directive 2008/105/EC) [59]. It is proven that low, subinhibitory concentrations of antibiotics in the environment have an influence on conjugal gene transfer, which is the main mechanism of horizontal gene transfer [62].

The assessment of hazards in a water environment is often critical to ensuring human health and public safety. For infection-treatment purposes, the examination of sources and characteristics of resistant-bacteria contamination of recreational water is very important. The inspection and monitoring of waterborne/water-based opportunistic pathogens should be connected with the quality criteria and regulatory documents.

## 4. Recreational Water Quality Law and Regulations

Almost 39% of the United States population, which is 50% of the global population, live near a coastal area [63]. In order for the water to be safe for humans, so its use through bathing areas does not endanger human health and life, it must meet a number of requirements. It is important to comply with the provisions on microbiological standards of recreational waters and to control the pathogenic microorganisms present, ensuring that the amount does not exceed the accepted limits and the places intended for bathing are completely safe for use. The United States Environmental Protection Agency (EPA) released quality criteria for recreational water in 1986 (U.S. EPA 2012). The aim of these criteria was to protect users, including swimmers, from exposure to water contaminated by microorganisms. Based on numerous studies and scientific publications, the EPA decided to revise those criteria in 2012 because of the visible link between illness and fecal contamination in recreational waters. They based this on *E. coli* and *Enterococcus* spp., as two bacterial indicators of fecal contamination [12,13]. Intestinal enterococci are commensal bacteria used in environmental monitoring such as water quality evaluation. They are present in high numbers in human and also in animal feces. Furthermore, they can be easily detected in contaminated water. As a result, enterococci are used as a fecal indicator organism (FIO), while their presence in water indicates possible long-standing fecal contamination. Concentrations of fecal-indicator bacteria in sand were higher than in samples of water [64]. Therefore, sand can potentially have a negative impact on water quality and may state a reservoir of fecal pollution. Intestinal enterococci are recommended by the WHO guidelines as the only regulatory parameter [6]. The European Parliament Directive from 2006, also called the Bathing Water Directive, requires the measurement of enterococci and *E. coli* at monitored recreational water sites [13,65]. *E. coli* is an innocuous resident of the gastrointestinal tract. Besides, it is abundant in human and animal feces, and it is a fecal contamination indicator [13]. Currently, there are no viral indicators in any of the recreational water regulations. The law regulations in most of the European countries are consistent with the Directive 2006/7/EC.

According to the European Parliament Directive 2006/7/EC and the Polish Ordinance of the Minister of Health from 2019 [66,67], surface water must meet the following requirements (Table 5). In contrast to defining the quality of surface water, the requirements that must be met by pool water in accordance with the Regulation of the Minister of Health of November 9 from 2015 (Table 6) are much more stringent and include more restrictive parameters that pool water must be subject to so it is usable [67]. When the quality of the water does not meet certain standards, measures to prevent bathing should be implemented, such as a ban on bathing or publication containing information warning people about bathing. Appropriate remedial action should also be taken by the bathing authority in question.

The bacterium can survive alone in different kinds of water (e.g., in brackish water), both independently (planktonic cell) or in biofilm form [51]. Many epidemiological studies show that recreational water users suffered from various illnesses with different localization in the body, e.g., upper respiratory tract infections, skin problems [28]. The biggest problem seems to be gastrointestinal illness. This is why water quality criteria have been formed all over the world [3]. WHO guidelines and standards elaborated by the European Union define a randomized control trial approach with culture methods, designed to assess microbial exposure [6].

There is no unified method or proper technique of opportunistic pathogen-identification or detection of the host origin of pathogens to encompass the sampling, assessment, and analysis of water for all microorganisms of interest [68,69]. Among others, there are many species and types of opportunistic pathogens, both non-culturable and culturable, with different growth conditions. The problem is no unified method, physical differences between the major bacteria and other pathogen groups, and low concentration of microorganisms in a large volume of sample. Besides the presence of contaminations and inhibitors from the water sample (including if it comes from natural reservoirs of water), established general scheme for water sample collection. Culture-dependent methods, which are used for pathogen detection in water, are limited by the excessive time needed to obtain reliable results and their low sensitivity. Furthermore, there is a lot of broad human pathogens that exist in a non-culturable state (e.g., *V. cholera)*. In culture-dependent and molecular methods, the monitoring of water quality and the source tracking using indicative pathogens have been selected. The false negative results may arise from this type of technique in consequence to the presence, for example, of a low number of pathogens in water samples. The water assessed, for example, by monitoring *E. coli,* as proper quality and pathogen-free, may be contaminated with viruses, fungi, or protozoa [69,70]. Moreover, there are no documents or they have only been distributed locally, that provide the criteria for assessing the proper quality of water sampling from natural sources only occasionally used for bathing or other recreational activities.

Currently, following of proper hygiene rules can reduce human exposure to pathogens [71]. The goal of this action is to reduce the possibility of human infection by fecal excreta that are not properly contained or treated. People can be exposed to pathogens through drinking contaminated water or eating contaminated food. It is important to ensure the safety of drinking water by keeping it free from fecal contamination. The wastewater should be collected and sent to a well-managed treatment plant. In fact, even 85–95% of collected wastewater is discharged to the environment without treatment in developing countries [71]. This action contributes to contamination of waterways, which impacts human health and the environment. Furthermore, reducing discharge of untreated wastewater to the environment would have a positive influence on people who earn their living using water, such as fishermen and farmers [71]. In the EU, re-recovery of water from usable wastewater accounts for a small percentage. European countries are taking numerous initiatives to re-circulate water. In the world, the lead in water recycling is the United States, which has proposed, as the first new approach to the reuse of wastewater, the transformation of treatment plants into plants that produce recovered resources from wastewater. European countries are constantly working on a common approach to reclaiming water for reuse. EU legal acts are to increase the scope of water protection against pollution and strive to internationalize the system in improving water quality [72]. In fact, there is a need to improve wastewater treatment infrastructure and implement better environmental protection policies to secure water quality [73]. The increase of water demand is caused by acceleration of population growth, land-use changes, and use of fertilizers. This led to progressive water degradation by widespread chemical contamination, hazardous cyanobacteria blooms, increased eutrophication, fecal contamination, and growing antibiotic resistance. Environmental impacts are worsening due to climate change, extreme weather, or precipitation events. It will be necessary to efficiently mitigate the influence of fecal wastes and sewage on water systems and human health in the future [73].

## 5. Conclusions

Aquatic environment and water sanitation systems are the key components of human health and antimicrobials, including antibiotics-resistance monitoring. Protection of water, especially drinking water, from contamination by human or other animal excrement in food processing wastes, sewage, and stormwater runoff is of paramount importance to everyone. Apart from fighting against pathogens, some steps should be taken to reduce chemical stressors (such as antibiotics, biocides, residual antimicrobials) in water environments and understand the different mechanisms of antibiotic-resistant genes spreading [74]. Of particular concern should be the better control of antibiotic doses used in animal breeding and in human curing, which lead to resistant strain selection. In ideal times, sanitation might be a critical point to help with the reduction of the antibiotic spread. New sanitation technologies should help with human and animal waste-streams, antibiotics, metals, and other agents to minimize their selective pressure actions. It is crucial to minimize human exposure to fecal pathogens and at the same time reduce the potential spread of resistant genes to human pathogens and natural environment microbiota. It is possible in some time to find characteristic genes that could act as pollution indicators. The European COST Action group works on a mixture of resistant-gene monitoring approaches. These studies are based on clinical and indicator genes, markers of gene transfer, as well as on cross-comparative analysis. Many researchers wait for results because they can create a new approach and possibilities.

There are several problems while conducting this kind of research. The best-known situation depicts gastrointestinal waterborne diseases. First of all, the verification of waterborne-infection severity is based on a self-report or literature data. This also includes information about start and end dates of symptoms. It could be problematic to recall such specific information, which has an impact on the accuracy of the duration estimates. Besides, there is a need to evaluate a factor to assess the severity of symptoms. Some of them, such as vomiting or diarrhea, are more serious when compared with nausea or stomachache. What is more, some symptoms correspond to pathogens with a longer incubation period than three days [5].

**Figure 1:**
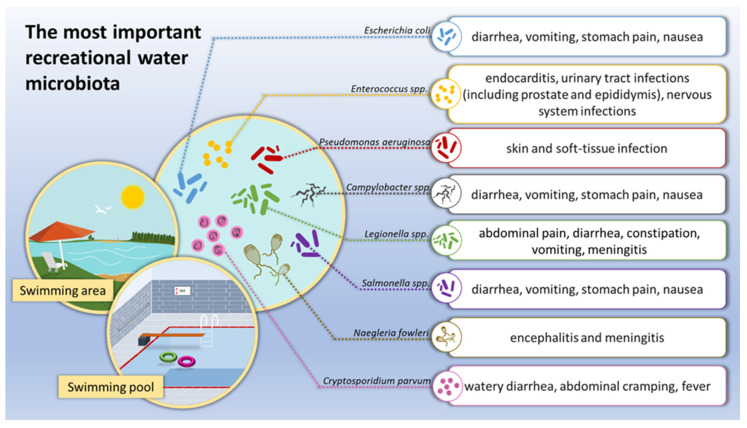
The most important microbiological threats in recreational waters.

As already known, recreational waters pose a threat to human health (Figure 1). This is why the quality of recreational waters is strictly monitored in many jurisdictions. Our review includes various opportunistic pathogens connected with environmental water, but it is limited by the relatively small number of available studies associated with natural water reservoirs only occasionally used for recreational activity. Most studies and regulatory documents are mainly focused on human-made and human-use water reservoirs. The largest assessments of illness risk are associated with official recreational activities and swimming places. However, increasing knowledge of disease-risk associated with recreational water used occasionally for human activities may prompt additional studies of water-based opportunistic pathogens and waterborne diseases. Moreover, the data must be expanded to include geographical and temporal variability of microorganisms including bacteria such as *E. coli* within a certain region and site-specific standards. The existence and persistence of opportunistic pathogens is greatly influenced by local biological, physical, and chemical water reservoir properties, including nutrient availability, temperature, and solar radiation.

Importantly, the risk for human health is poorly quantified and may be exacerbated by various mechanisms and antibiotic-resistance factors that require further understanding. Reducing such a risk by taking specific actions seems to be desirable in newer solutions. It is important to take care and further research this problem, as more and more people find water activities attractive.

## Figures and Tables

**Table 1:** Examples of pathogens found in recreational water on the basis of literature data.

Species	Category	Literature Source
*Legionella* spp.	water-based	[28]
*Salmonella* spp.	waterborne	
*Vibrio vulnificus*	waterborne/water-based	
*Mycobacterium marinum*	water-based	
*Edwardsiella tarda*	waterborne	
*Burkholderia pseudomallei*	water-based	
*Aeromonas spp.*	waterborne/water-based	
*Campylobacter spp.*	waterborne	
*other Gram-negative bacilli/rods* *	waterborne/water-based	
*Leptospira spp.*	water-based	
*Schistosoma spp.*	water-based	
*Giardia duodentalis*	waterborne	
*Naegleria fowleri*	water-based	
*Cryptosporidium*	waterborne	[29]
*Cyanobacteria*	waterborne/water-based	[30,31]
*Legionella pneumophila*	water-based	[4]
*Mycobacterium avium complex*		
*Pseudomonas aeruginosa*		
*Aeromonas* spp.	waterborne	[32]
Enteroinvasive Escherichia coli (EIEC)	water-based	[33]
Escherichia coli O157:H7		
Shigella spp.		
*Salmonella* spp.	waterborne	[34]

* other Gram-negative bacilli/rods—Gram-negative bacteria include the Enterobacteriacae family (Escherichia coli, Klebsiella pneumoniae, Enterobacter aerogenes, Enerobacter cloacae, Serratia marscescens, Proteus mirabilis, Proteus vulgaris, Citrobacter freundii and others), Pseudomonas aeruginosa, Acinetobacter baumannii, Stenotrophomonas maltophilia and Burkholderia cepacia. Different bacteria (waterborne and water-based) belong to this genus.

**Table 2:** Infectious doses of pathogens on the basis of literature data.

Pathogen	Infectious Dose	Literature
*Cryptosporidium parvum*	87 oocysts	[29]
*Salmonella typhi*	10^5^ cells	[35]
*Salmonella typhimurium*	10^9^ cells	
*Campylobacter jejuni*	500 cells	
Enterohaemorrhagic *E. coli* (EHEC) O157:H7	10–100 cells	
Enterohaemorrhagic *E. coli* (EHEC)	10^2^–10^6^ cells	[37]
Enteroinvasive *E. coli* (EIEC)	10^6^–10^10^ cells	
Enterotoxigenic *E. coli* (ETEC)	10^8^–10^10^ cells	
Enteropathogenic *E. coli* (EPEC)	10^8^–10^10^ cells	

**Table 3:** Clinical cases of patients infected via contaminated water with treatment data.

Disease/Ailment	Etiological Factor	Source of Microorganisms	Number of Cases	Treatment/Results	Literature
wound infection	*Aeromonas caviae*	trauma due to foreign body to the foot	1	treatment: amoxicillin with clavulanic acid (deterioration) hospitalization: ceftriaxone, amikacin full recovery	[44]
necrotizing soft-tissue infection	*Aeromonas* spp.	contaminated water	15 *A. hydrophila*-10 *A. sobria*-4 *A. caviae*-1	treatment: ceftriaxone or ceftazidime combined with doxycycline or gentamicin results: 5 limb amputations, sepsis, skin lesions, hypotension; mortality rate: 27%	[45]
skin and soft-tissue infection	*Aeromonas* spp.	contamination of water after tsunami wave	145 *A. hydrophila*-104 *A. veronii* biovar sobria-41	most cases resistant to amoxicillin-clavulanate and first-generation cephalosporins	[46]
	*Escherichia coli*		116	susceptible to antibiotics	
	*Klebsiella pneumoniae*		93	susceptible to antibiotics	
	*Proteus* spp.		47 *P. vulgaris*-27 *P. mirabilis*-20	most cases resistant to amoxicillin-clavulanate and first-generation cephalosporins	
	*Pseudomonas aeruginosa*		77	most cases resistant to amoxicillin-clavulanate and first-generation cephalosporins	
	*Staphylococcus* spp.		17 *S. aureus*-11 (including 2 MRSA cases) CoNS-6	empirical therapy was undertaken-amoxicillin-clavulanate and first-generation cephalosporins	
pneumonia	*Legionella pneumophila* serogroup 1 Pontiac Philadelphia ST899	contaminated water supply network	277	not mentioned	[47]
pneumonia	*Legionella* spp.	fresh water	-	quinolone	[48]
multiple complex deep lacerations right leg	*Pseudomonas* *fluorescens;* *Klebsiella* *pneumoniae*	fresh water- fall and struck by a propeller of a motorboat	1	hospitalization: piperacillin-tazobactam, vancomycin	[49]
	*Candida tropicalis*			hospitalization: cefazolin, gentamicin	
leg lacerations, foot open fractures	*Staphylococcus aureus* (MSSA)	fresh water-wakebording	1	hospitalization: cefazolin, gentamicin, ciprofloxacin	
forearm lacerations	*Staphylococcus aureus* (MSSA)	fresh water-fall from a jet ski	1	hospitalization: clindamycin, gentamicin	
legs and back multiple lacerations	*Acinetobacter baumannii,* MRSA, MSSA	fresh water	1	hospitalization: piperacillin-tazobactam	
watery diarrhea, abdominal cramping, fever	*Cryptosporidium*	water in a swimming pool	5	2 patients required hospitalization	[50]

CoNS-coagulase-negative staphylococci, MSSA-methycyllin-sensitive *Staphylococcus aureus*; MRSA-methycyllin-resistant *Staphylococcus aureus*.

**Table 4:** Microorganisms spread directly or indirectly by water.

Microorganism	Source of Microorganism	Presented Antibiotic Resistance	Literature
*Aeromonas* spp.	municipal wastewater system	over 72% classified as multidrug resistant most isolates were resistant to beta-lactams, tetracyclines and aminoglycosides	[56]
*Serratia marcescens*	aerated filter system of onsite wastewater treatment facility	loss of sensitivity for 5 antibiotics: lomefloxacin, enoxacin (fluoroquinolones), nalidixic acid (quinolone), paromomycin (aminoglycoside), novobiocin	[57]
*Escherichia coli*	recreational water	53% strains resistant to ampicillin 56% strains resistant to ticarcillin	[55]
*Escherichia coli*	water and contaminated artificial snow	74.19% *E. coli* isolated from snow were resistant to ampicillin and 51.61% isolates to amoxicillin-clavulanate 45% of isolates classified as multidrug resistant (MDR)	[58]
*Enterococcus faecalis*, *Enterococcus faecium*, Gram- negative rods	river	13% of *E. faecalis* isolates resistant to fluoroquinolones, tetracyclines, aminoglycosides, 31% of *E. faecium* resistant to beta-lactams, fluoroquinolones, tetracyclines and aminoglycosides, nearly 50% of Gram-negative isolates resistant to beta-lactams	[59]

**Table 5:** The requirements for surface water [66].

Parameter	Excellent Quality	Good Quality	Sufficient Quality
Intestinal enterococci (cfu/100 mL)	200 *	400 *	330 **
*Escherichia coli* (cfu/100 mL)	500 *	1000 *	900 **

* based upon a 95th percentile evaluation; ** based upon a 90th percentile evaluation; cfu-colony forming units.

**Table 6:** The requirements for swimming pool waters [67].

The Highest Permitted Number of Microorganisms (cfu—Colony Forming Units or MPN—the most Probable Number)					
Parameter	Water Introduced into the Pool Basin from the Circulation System ^a^	Water in the Pool Basin *	Water in Swimming Pool Basins Equipped with Devices Generating Water-air Aerosol ^b^	Water in Pool Basins Made Available for Swimming Lessons for Infants and Toddlers up to 3 Years of Age	Water in Showers
*Escherichia coli* per 100 mL of water	0	0	0	0	–
*Pseudomonas aeruginosa* per 100 mL of water	0	0	0	0	–
Total number of microorganisms at 36 ± 2 °C after 48 h per 1 mL of water **^c^**	20	100	100	100	–
Coagulase positive staphylococci per 100 mL of water	–	–	–	0	–
*Legionella* spp. per 100 mL of water	0	0 ^d^	0	0	<100 ^e^

^a^ applies to: fresh water (surface water or groundwater meeting the requirements specified in the regulations for drinking water), salt water (including marine and brine water containing from 5 g/L to 15 g/l of minerals (mainly chlorides) and thermal water (groundwater, which at the outflow from the intake have a temperature of not less than 20 °C (excluding water from drainage of mining excavations).; ^b^ the test water sample should be collected from the swimming pool basin, at the shortest possible distance from the nozzle outlet.; ^c^ does not apply to outdoor swimming pools.; ^d^ the test should be performed when the water temperature is ≥ 30 °C.; ^e^ should be tested in hot water systems. The test sample should be taken from at least 1 in 10 showers. * including paddling pools for children’s games.

## Data Availability

Not applicable.
